# Medical Management of Crohn's Disease

**DOI:** 10.7759/cureus.8351

**Published:** 2020-05-29

**Authors:** Ajay K Gade, Nathan T Douthit, Erin Townsley

**Affiliations:** 1 Internal Medicine, Brookwood Baptist Medical Center, Birmingham, USA; 2 Medical Education Internal Medicine, Brookwood Baptist Health, Birmingham, USA; 3 Medical Education Internal Medicine, Brookwood Baptist Medical Center, Birmingham, USA

**Keywords:** crohn's disease

## Abstract

Crohn's disease (CD) is a chronic inflammatory bowel disease that can affect the entire gastrointestinal tract from the mouth to the anus, sparing the rectum. The goal of medical therapy is to induce remission with medications, followed by the administration of maintenance medications to prevent a relapse of the disease. The concept of induction of remission and maintenance of remission is very important, as there is an overlap of medications used to induce and maintain remission. Physicians first direct treatment to induce a remission that involves relief of symptoms and mucosal healing of the lining of the colon and then provide long-term treatment to maintain the remission. Standard treatment for CD depends on the extent of involvement and disease severity, for example, mild, moderate, severe, and fulminant.

## Introduction and background

Crohn’s disease (CD) is an idiopathic inflammatory disorder. It is influenced by genetics, the environment, and immunologic milieu. The incidence of this disease is increasing and treatment options are always evolving. The treatment options include medications, nutrition supplements, surgery, or a combination of these [1]. The goals of treatment are to control inflammation, correct nutritional deficiencies, and relieve symptoms like abdominal pain, diarrhea, and rectal bleeding. Treatment for CD depends on the location and severity of the disease, complications, and response to previous medical treatment when treated for recurring symptoms. Some people have long periods of remission, sometimes years, when they are free of symptoms [2]. However, the disease usually recurs at various times over a person’s lifetime. This article reviews the pharmaceutical options available for the management of CD.

## Review

Pharmacotherapy agents

The pharmacologic treatment of CD involves a wide array of agents. These agents have varying indications and mechanisms of action. They can be classified into five groups: aminosalicylates, corticosteroids, immunosuppressive agents, antibody agents, and antibiotics [3].

Aminosalicylates

Aminosalicylates are a class of drugs that deliver the active component, mesalamine, to target tissues. Aminosalicylates are used in the management of CD by an anti-inflammatory effect on the intestine. The drugs in this class include sulfasalazine, olsalazine, and mesalamine. 5-aminosalicylic acid (5-ASA) and mesalazine are the therapeutically active compounds in sulfasalazine [4-5]. The efficacy and side effects of these medications can be seen in Table 1.

**Table 1:** Aminosalicylates, efficacy, and side effects CD: Crohn's disease

Aminosalicylates	Efficacy	Side effect
Sulfasalazine	Can be used for both active disease and maintenance in both mild or moderate CD	Headache, Steven-Johnson Syndrome (SJS), oligospermia, hepatotoxicity, and hemolytic anemia
Mesalamine	Can be used for both active disease and maintenance in both mild or moderate CD	Watery diarrhea and interstitial nephritis
Olsalazine	Used to treat mild or moderate CD	Headache, nausea, vomiting, hepatotoxicity, and anorexia

Corticosteroids

The commonly used corticosteroids are cortisone, prednisone, prednisolone, hydrocortisone, methylprednisolone, beclometasone, and budesonide. Corticosteroids reduce inflammation and induce the remission of active CD. They are commonly prescribed when 5-ASA compounds are ineffective. These agents work by suppressing interleukin transcription and arachidonic-acid metabolism and by stimulating apoptosis of lymphocytes in the gut [6]. The side-effect profile is similar for all these agents and includes Cushing features, acne, weight gain, and dyspepsia, which also can lead to acute adrenal insufficiency if withdrawn abruptly. Other side effects include hypertension, diabetes, and osteoporosis [7].

Immunosuppressive Drugs

Due to immunological influence, there is a substantial role for immunomodulatory agents in CD. These agents have varying efficacy and indications. The commonly used immunosuppressants are 6-Mercaptopurine, azathioprine, methotrexate, and tacrolimus [8].

Azathioprine (AZA)/6-Mercaptopurine(6-MP)

AZA is a prodrug of 6-MP. The goal of treatment with AZA/6-MP is to prevent flare-ups, reduce the need for corticosteroids, improve quality of life by controlling diarrhea, gastrointestinal bleeding, and pain. These agents are often used for maintenance therapy of CD or AZA/6-MP and are also effective for maintaining a corticosteroid-induced remission [9]. AZA/6-MP does not offer any additional advantage over the placebo in inducing remission in the treatment of CD, as these medications can take up to three months to achieve a clinical response. However, these medications can be used for the maintenance of remission. Pancreatitis, hepatotoxicity, and bone marrow suppression are the reported adverse effects in patients using AZA/6-MP [10].

Methotrexate

Methotrexate (MTX) inhibits the dihydrofolate reductase enzyme involved in folic acid metabolism with the subsequent inhibition of the synthesis of deoxyribonucleic acid (DNA), ribonucleic acid (RNA), and protein and the inhibition of folate enzyme-dependent immunomodulation [11]. MTX is an effective alternative for patients with CD who have failed other immunosuppressive drugs. It is used for maintenance therapy since it takes up to 12 weeks to achieve clinical response. Its use is limited by uncommon but serious side effects such as hepatotoxicity, leukopenia, and pneumonitis [12].

Cyclosporine

Cyclosporine is an immunomodulator that works by inhibiting the production of cytokines, thereby regulating T-cell activation [13]. Intravenous cyclosporine is an effective therapy for perianal, rectovaginal, and enterocutaneous fistulas in CD. The oral form of cyclosporine is not useful for maintaining long-term beneficial effects as opposed to the intravenous form. The adverse effects of cyclosporine include nephrotoxicity, hypertension, hypomagnesemia, tremor, gingivitis, and hirsutism [12,14].

Antibody Treatment

CD patients produce too much tumor necrosis factor-alpha (TNF-alpha), a protein that helps regulate immune cells and inflammation in the body. Disproportionate TNF-alpha can cause exaggerated immune activation, leading to intestinal inflammation and signs and symptoms of CD [15]. Anti-TNF agents (infliximab, adalimumab, certolizumab pegol) should be used to treat CD that is resistant or refractory to corticosteroids, AZA/6-MP, or methotrexate. Infliximab and adalimumab are preferred for induction of remission while all three anti-TNF agents are used to maintain remission [16]. The combination therapy of infliximab with immunosuppressants is more effective than treatment with either immunosuppressants alone or infliximab alone in patients who are naive to those agents. The Effect of Tight Control Management on Crohn's Disease (CALM) trial is the first study in patients with early CD, to show the well-timed escalation and de-escalation of anti-TNF therapy on the basis of symptomatic improvement combined with an objective assessment of biomarkers of inflammation such as C-reactive protein and fecal calprotectin, resulting in a better outcome both clinically and endoscopically than considering clinical improvement alone [17].

Infliximab

Infliximab is an intravenously administered agent used to treat moderate to severely active CD and active fistulising CD in adults. It can reduce signs and symptoms and induce and maintain remission in adult patients with moderately active to severely active Crohn's disease who have not responded well to other therapies [18]. The common adverse effects of infliximab include respiratory infections, headache, cough, and gastritis. Infusion reactions can occur up to two hours after the administration, which includes fever, chills, shortness of breath, chest pain, rashes, and fluctuations in blood pressure [19].

*Adalimumab* 

Adalimumab is used in the treatment of moderate to severe CD, to induce and maintain clinical remission in adults who have lost response to treatment or are intolerant to infliximab. One of the major advantages of adalimumab and certolizumab over infliximab is the subcutaneous route of administration, as it is more convenient for patients with better compliance [20]. Adverse effects of adalimumab include serious infections such as tuberculosis (TB), bacterial sepsis, invasive fungal infections, such as histoplasmosis, and other opportunistic infections. Prior to administering adalimumab, the patients should be tested for latent TB. These patients should also be monitored for active TB during treatment, regardless of the initial latent TB test results. Lymphoma and other malignancies have been reported in the pediatric age group. Post-marketing surveillance detected hepatosplenic T-cell lymphoma, a rare type of T-cell lymphoma more common in patients treated with this agent [21].

Certolizumab Pegol

Certolizumab pegol is currently the only PEGylated anti-TNFα approved for the treatment of CD and rheumatoid arthritis. The humanized antigen-binding fragment (Fab) of a monoclonal antibody conjugated to polyethylene glycol increases the half-life of the certolizumab, decreasing the dosage and frequency of drug administration [22]. It is reserved for second or third-line anti-TNF agents in patients with CD who responded to infliximab or adalimumab and then lose response or become intolerant. The side-effect profile is similar to the other antibody-derived agents, such as the increased risk of infections like TB and other opportunistic infections [23].

Ustekinumab

In September 2016, the United States Food and Drug Administration approved the use of ustekinumab for use in CD. It is an (interleukin) IL-12 and IL-23 antagonist. It is used in patients with moderate to severe CD who have failed treatment with corticosteroids or immunosuppressants [24]. The adverse effects of using ustekinumab are anaphylactic reactions, diarrhea, upper respiratory tract infections, and injection site reactions [19,24].

Anti-Integrin Agents

Anti-integrin agents can be used to induce and maintain remission in patients with CD. They are used for maintaining remission in patients whose remission is induced by corticosteroids; they are also used in patients resistant or refractory to anti-TNF agents. Natalizumab and vedolizumab are the two anti-integrin agents approved for the treatment of CD. Natalizumab is associated with progressive multifocal leukoencephalopathy (PML) [25].

Antibiotics

Antibiotics can be used to manage mild to moderate CD associated with fistulas and abscesses or unresponsive to aminosalicylates. Metronidazole and ciprofloxacin have been implicated in the treatment of CD. Metronidazole can cause disulfiram-like reactions, candidiasis, Steven-Johnson Syndrome (SJS), thrombophlebitis, and neutropenia. Ciprofloxacin can cause prolonged QTc intervals, tendonitis or tendon rupture, peripheral neuropathy, photosensitivity, and Clostridium (C.) difficile diarrhea [19,26].

Goals of treatment and disease activity

CD is classified based on severity (Tables 1-2) The goal of medical therapy is to induce remission with medications, followed by maintenance with medications to prevent a relapse of the disease. Disease activity can be classified by several scoring systems (Tables 2-3) [27].

**Table 2:** Classification of CD based on severity CD: Crohn's disease

Status	CDAI	Description from ACG Guidelines
Remission	< 150	Asymptomatic or without any symptomatic inflammatory sequelae
Mild to moderate	150 to 220	Ambulatory and able to tolerate oral alimentation without manifestations of dehydration, systemic toxicity, abdominal tenderness, painful mass, intestinal obstruction, or > 10% weight loss
Moderate to severe	220 to 450	Failed to respond to treatment for mild to moderate disease, or those with more prominent symptoms of fever, significant weight loss, abdominal pain or tenderness, intermittent nausea or vomiting, or significant anemia
Severe	> 450	Persistent symptoms despite the introduction of conventional corticosteroids or biologic drugs as outpatients, or individuals presenting with high fevers, persistent vomiting, evidence of intestinal obstruction, significant peritoneal signs such as involuntary guarding or rebound tenderness, cachexia, or evidence of an abscess

**Table 3:** The Crohn's Disease Activity Index or CDAI is a tool used to quantify the symptoms of patients with Crohn's disease [27]

Clinical or laboratory variable	Weighting factor
Number of liquid or soft stools each day for seven days	2
Abdominal pain (graded from 0-3 on severity) each day for seven days	5
General well being, subjectively assessed from 0 (well) to 4 (terrible) each day for seven days	7
Presence of complications	20
Taking atropine/diphenoxylate or opiates for diarrhea	30
Presence of an abdominal mass (0 as none, 2 as questionable, 5 as definite)	10
Absolute deviation of hematocrit from 47% in men and 42% in women	6
Percentage deviation from standard weight	1

The concept of the induction and maintenance of remission is very important. There is some overlap of medications used to induce and maintain remission, but the treatments are different. The initial treatment is directed towards inducing a remission that involves relief of symptoms and mucosal healing and then followed by long-term treatment to maintain remission [28]. Standard treatment for CD depends on the extent of involvement and disease severity such as mild, moderate, severe, and fulminant [27].

Management of mild to moderate CD

Ileitis and Colitis

**Figure 1:**
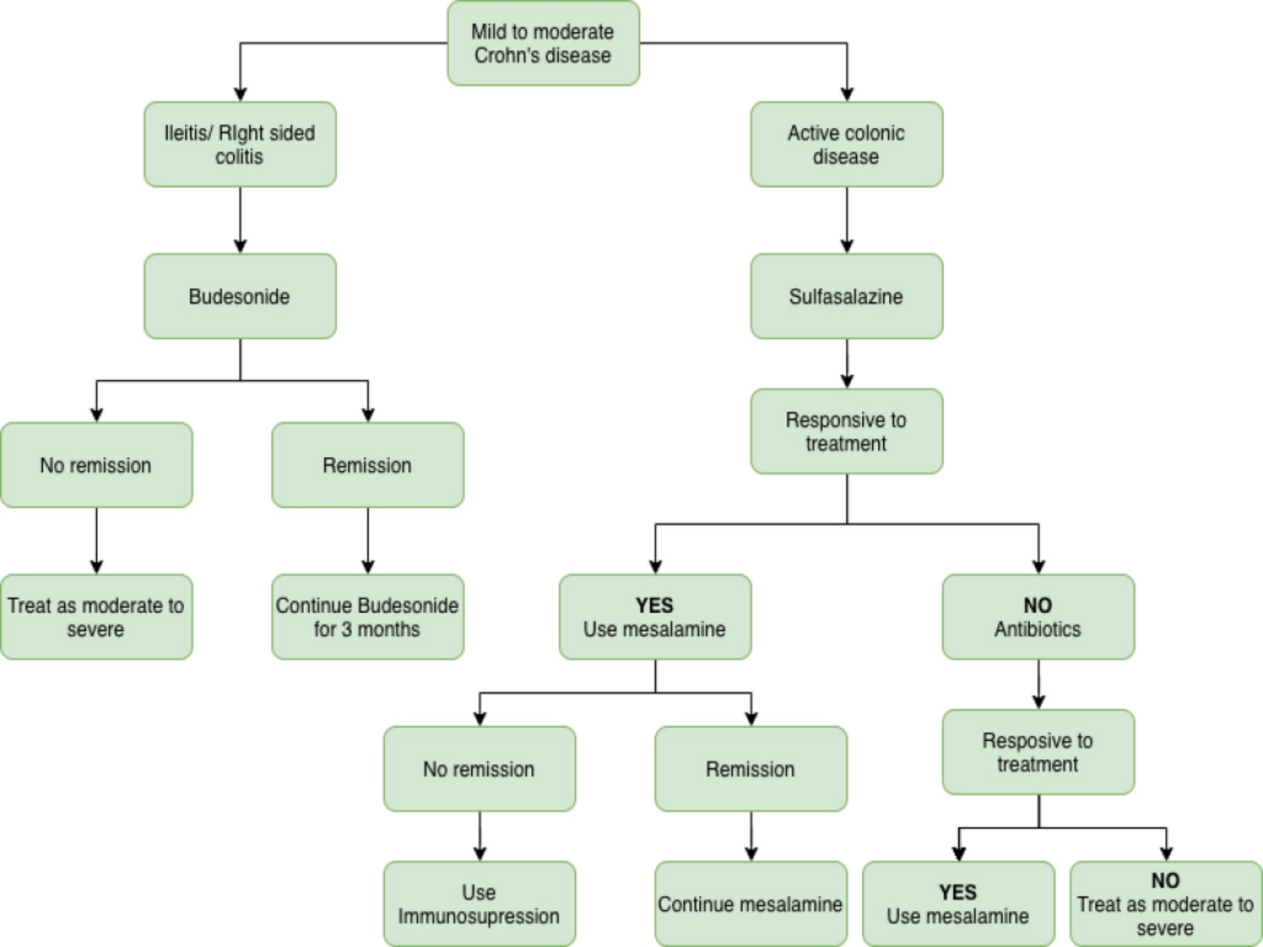
Mild to moderate CD CD: Crohn's disease

Oral budesonide 9 mg/day is used for the treatment of ileitis and right-sided colitis. If the patient responds to the treatment, budesonide is continued with tapering for a period of three months. If the ileitis/right-sided colitis recurs, restart the budesonide at a higher dose and taper it over three months or treat it as moderate to severe CD (Figure 1) [29-32]. If the symptoms do not recur, a follow-up colonoscopy is scheduled within a year.

Active Colonic Disease

In patients with active colitis, sulfasalazine is used for inducing remission, followed by mesalamine for maintenance. If remission is achieved, continue the mesalamine; if not, use immunosuppressants. If the patient is not responding to the sulfasalazine, use antibiotics (metronidazole/ciprofloxacin) for the induction of remission [31-32]. If this is successful, use mesalamine for maintenance therapy. In patients not responding to sulfasalazine and antibiotics, treat it as a moderate to severe disease (Figure 1) [33].

Oral Lesion

The preferred agents to treat oral lesions include triamcinolone acetonide, mesalamine, sulfasalazine, and balsalazide. AZA/6-MP and infliximab can be used as an alternative regimen. A combination of oral mesalamine and mesalamine enema twice weekly is more effective than oral treatment alone [31-32].

Gastroduodenal Disease

Gastroduodenal involvement is a rare manifestation of CD. Intense acid suppression with a proton pump inhibitor, H2 antagonists, and sucralfate can be used to treat gastroduodenal disease. Oral mesalamine can also be used. Initial treatment for active gastroduodenal CD often involves corticosteroids along with a proton pump inhibitor. AZA/6-MP has been shown to maintain corticosteroid-induced remission and should be instituted early in the disease course. Balloon dilation is used to treat strictures in these patients. Surgical intervention should be considered in patients with massive persistent gastrointestinal bleeding, gastric outlet or duodenal obstruction, fistula, or abscess [34].

Management of moderate to severe CD

Acute Management

**Figure 2:**
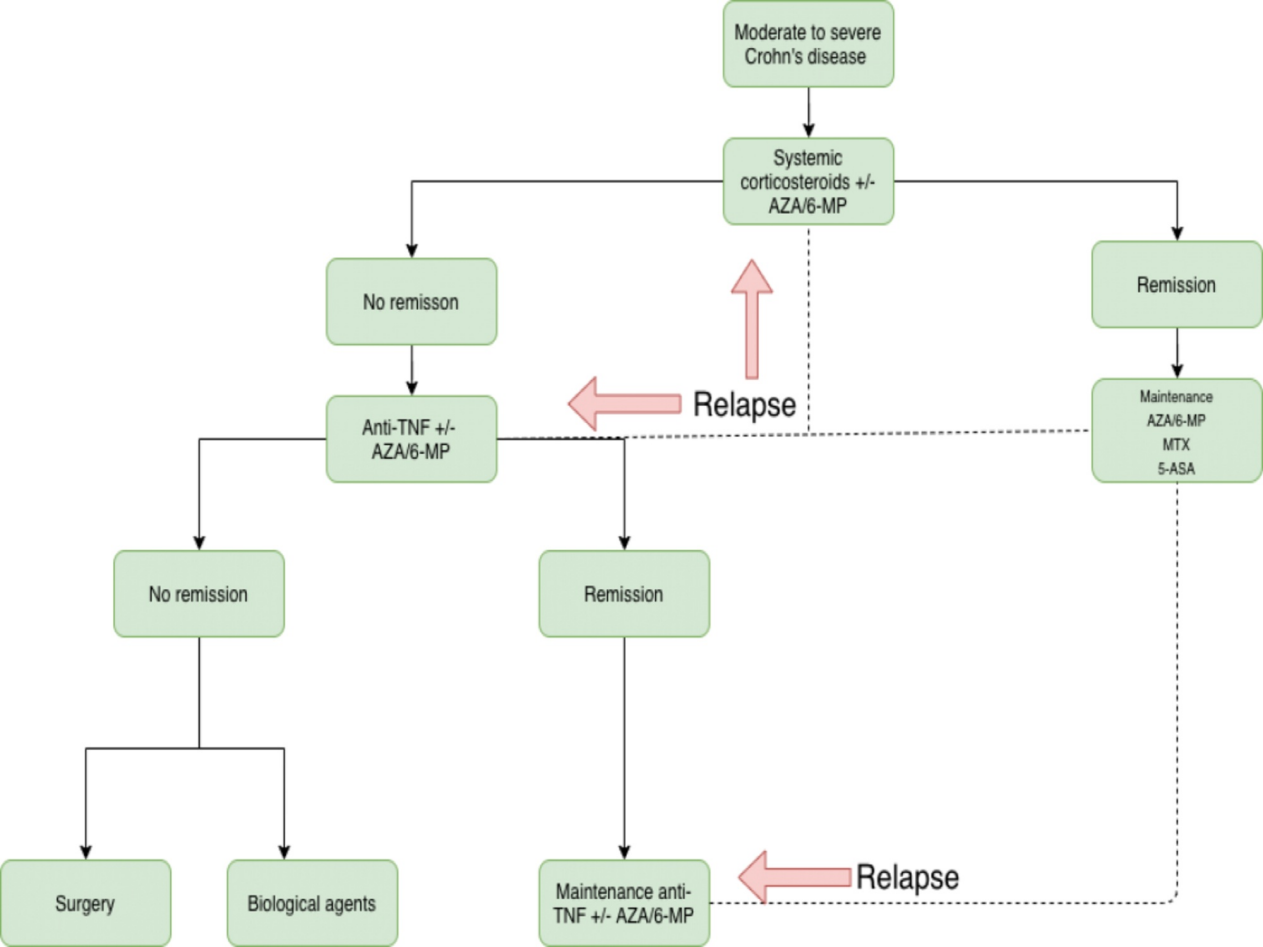
Moderate to severe CD CD: Crohn's disease

In patients with moderate to severe CD refractory to aminosalicylates in combination with topical therapy, oral steroids are used. If they are refractory to oral steroids, methotrexate can be used alternatively. Other alternative regimens include infliximab, adalimumab, certolizumab, and azathioprine. Infliximab is contraindicated in patients with untreated latent tuberculosis, pre-existing demyelinating disorder, optic neuritis, moderate to severe heart failure, and current malignancy (Figure 2) [31]. 

Maintenance of Remission

For maintenance of remission in moderate to severe CD, 6-mercaptopurine or azathioprine is preferred. Infliximab can be used in patients with successful induction with infliximab. The alternate regimen includes infliximab and azathioprine therapy, methotrexate therapy for methotrexate-induced remissions, adalimumab therapy for adalimumab-induced remissions, certolizumab pegol therapy for certolizumab pegol-induced remissions, and natalizumab therapy for natalizumab-induced remissions. Corticosteroids are not preferred for long-term maintenance therapy. Complete blood counts should be checked every three months when using immunosuppressants [9].

Corticosteroids

Oral corticosteroids are often used for symptom management of moderate to severely active CD. Corticosteroids are used from 10- 40 mg/day, and not used to maintain remission, as they cannot help in mucosal healing and there is a major concern for the long-term side adverse effects of the use of steroids. Patients are usually treated for two to three months to induce remission and once a clinical response is achieved, the steroids are tapered [6-7,32].

Immunosuppressants

AZA/6-MP may take three to four months to demonstrate clinical response and are, therefore, used for the maintenance of remission rather than induction [8]. These medications should be considered in patients with steroid dependence or resistant to other forms of treatment. The US Food and Drug Administration (FDA) recommends thiopurine methyltransferase (TPMT) genotyping or phenotyping before initiating thiopurines as it allows patients with increased risk for toxicity to be identified and a dose of thiopurines is adjusted or alternative treatment can be assessed [35]. Methotrexate is effective and should be considered for use in alleviating signs and symptoms in patients with steroid-dependent CD for maintaining remission [12].

Anti-TNF Agents

Anti-TNF agents, such as infliximab, adalimumab, and certolizumab pegol, are taken up to two weeks to show clinical response and, therefore, can be used to induce and maintain remission in moderate to severe CD. These agents should be used in managing CD that is refractory to corticosteroids or immunosuppressants. In patients who are treatment-naive to infliximab and thiopurines, studies have shown them combining these two drugs have a superior response than the use of individual drugs separately [15-17].

Agents Targeting IL-12/23 (Anti-p40 Antibody)

Ustekinumab is used to treat moderate-to-severe CD patients who have previously failed treatment on steroids, AZA/6-MP, methotrexate, or anti-TNF agents. It is also used in patients naive to anti-TNF agents [19,24].

Anti-Integrin Agents

For patients with moderate to severe CD, vedolizumab with or without immunosuppressants is more effective than a placebo and should be considered for the induction of symptomatic remission in patients with CD. Natalizumab is more effective than a placebo and should be considered for the induction of symptomatic response and remission in patients with active CD. Natalizumab should be used for the maintenance of natalizumab-induced remission of the CD only if serum antibody to John Cunningham (JC) virus is negative due to the risk of progressive multifocal leukoencephalopathy [27,36].

Management of severe to fulminant Crohn's disease

Acute Management

**Figure 3:**
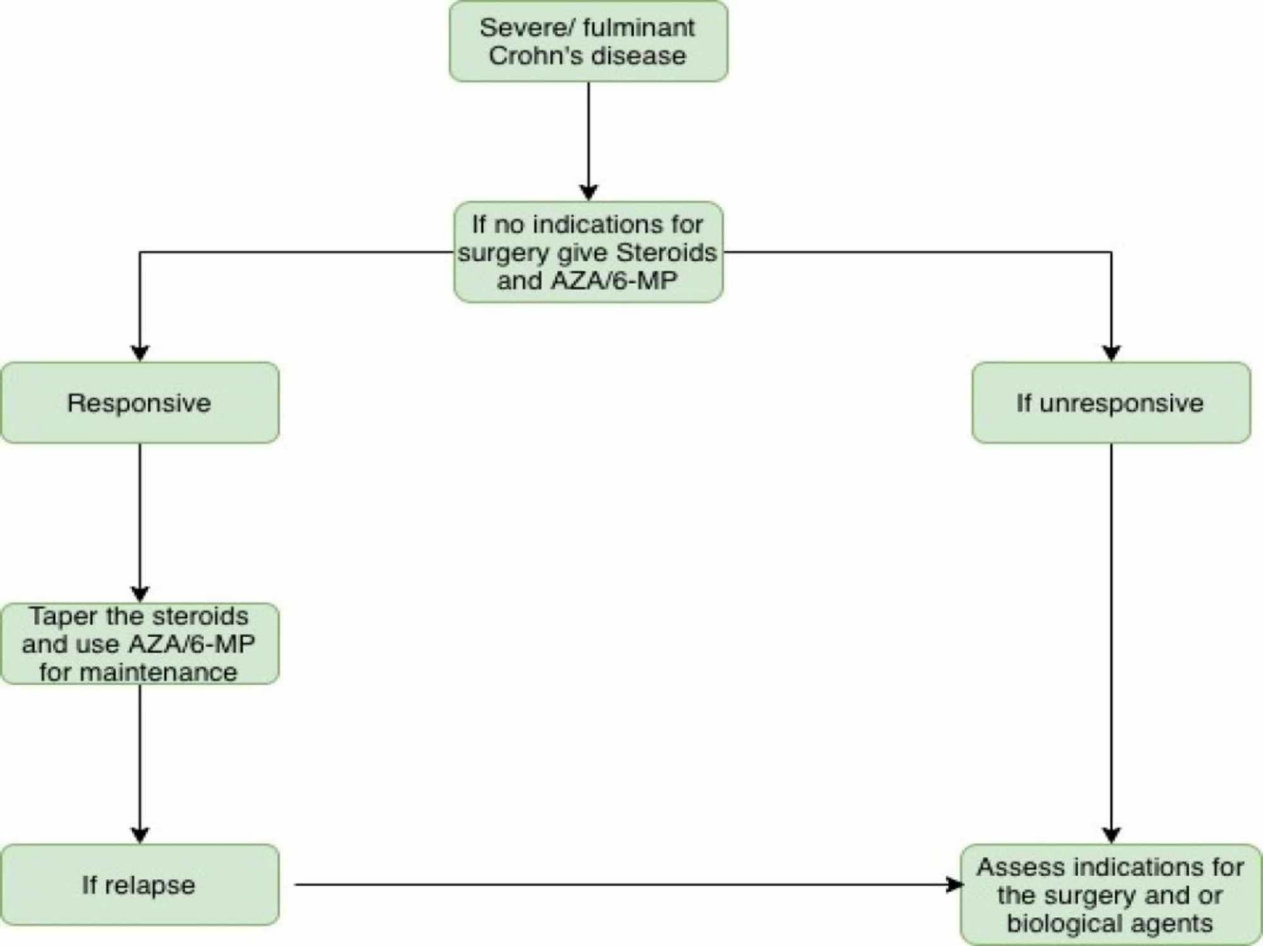
Severe or fulminant CD CD: Crohn's disease

The preferred regimen for the acute management of the severe to fulminant CD is maximal oral treatment with prednisone and oral aminosalicylate drugs and topical mesalamine [37]. Alternatively, infliximab 5 mg/kg can be used if the patient is refractory to the above regimen. Intravenous corticosteroids should be used to treat severe or fulminant CD [6]. Failure to show significant improvement within a week is an indication for colectomy. Infliximab can be effective in dodging colectomy in patients unresponsive to corticosteroids. AZA/6-MP is preferred for maintaining remission (Figure 3) [17].

Management of the complications of CD

Small Bowel Obstruction (SBO)

SBO in CD responds to medical therapy, such as bowel rest, nasogastric suction, and steroids. SBO from stenosis secondary to strictures/adhesions may require surgical therapy as medical therapy in these patients can result in recurrent obstruction [38].

Fistula

The two types of fistulas are internal and external fistulas. Internal fistulas include enteroenteric, gastrocolic, duodenocolic, rectovaginal, and rectovesical. External fistulae terminate on the skin, perianal surface, or stoma and are typically associated with pain and discharge [39]. Metronidazole and ciprofloxacin are considered first-line therapies in the management of perianal fistulae in CD. Oral corticosteroids are contraindicated, as they can cause or exacerbate the abscess formation. Immunosuppressants are effective for perianal fistula disease but noticeable improvement is slow and healing is often incomplete [10]. Recurrence and exacerbation are common after the discontinuation of drugs.

**Table 4:** The Montreal classification is used to describe the age, location/extent, and behavior of CD [40] CD: Crohn's disease

Age at diagnosis	A1: <16 years; A2: 17-40 years; A3: >40 years.
Location	L1: ileal; L2: colonic; L3: ileocolonic; L4: isolated upper digestive
Behavior	B1: non-stricturing, non-penetrating; B2: stricturing; B3: penetrating; B4: perianal disease

## Conclusions

CD is characterized by chronic, episodic inflammation of the entire gastrointestinal tract from the mouth to the anus, leading to poor quality of life in these patients. Treatment of CD involves the induction and maintenance of remission. The therapeutic options available are aminosalicylates, corticosteroids, immunosuppressants, biologic agents, antimicrobials, and newer generation medications such as ustekinumab and anti-integrin agents. A few patients are not completely responsive to traditional treatment or lose efficacy over the years; in these patients, new treatment drug options might offer some hope in achieving remission for the long term with lower relapse rates.
